# Combined active and passive heat exposure induced heat acclimation in a soccer referee before 2014 FIFA World Cup

**DOI:** 10.1186/s40064-016-2298-y

**Published:** 2016-05-13

**Authors:** A. D. Ruddock, S. W. Thompson, S. A. Hudson, C. A. James, O. R. Gibson, J. A. Mee

**Affiliations:** A016 Collegiate Hall, Centre for Sport and Exercise Science, Sheffield Hallam University, Sheffield, South Yorkshire S10 2BP UK; Academy of Sport and Physical Activity, A209 Collegiate Hall, Sheffield Hallam University, Collegiate Crescent Campus, Eccelsall Road, Sheffield, S10 2BP UK; Department of Sport, Health and Nutrition, Leeds Trinity University, Brownberrie Lane, Horsforth, Leeds, LS18 5HD UK; Environmental Extremes Laboratory, Centre for Sport and Exercise Science and Medicine (SESAME), University of Brighton, Welkin Laboratories, Denton Road, Eastbourne, East Sussex BN20 7SR UK; Centre for Sports Medicine and Human Performance (CSMHP), Heinz Wolff Building, Brunel University London, Uxbridge, UB8 3PH UK; School of Sport and Health Sciences, George Building, Bangor University, Bangor, UK

**Keywords:** Interval training, Core temperature, Exercise, Performance, Plasma volume, Thermoregulation, Hyperthermia, Aerobic

## Abstract

**Introduction:**

The 2014 FIFA World Cup was held in Brazil, where the climatic conditions presented a significant thermoregulatory and perceptual challenge to those unfamiliar with the heat and humidity.

**Case presentation:**

This case report documents the adaptation induced by a novel mixed methods (isothermic and passive) heat acclimation (HA) regime for a northern European professional soccer match official prior to the tournament. The intervention involved 13 HA sessions over an 18 day period comprising five isothermic HA sessions whereby intermittent running was used to target and maintain tympanic temperature (Ty_temp_) at 38 °C for 90 min, and seven passive HA sessions of 48 °C water bathing for 30 min. The athlete performed a heat stress test (HST) (35 min running at four incremental intensities in 30 °C) and a repeated high-intensity running test (as many 30 s self-paced efforts as possible, to a maximum of 20, with 30 s passive recovery) before and after the intervention. The mixed methods HA regime increased plasma volume (+7.1 %), and sweat loss (+0.9 L h^−1^), reduced exercising Ty_temp_ (−0.6 °C), and mean body temperature (−0.5 °C). High-intensity running performance improved after HA (+29 %), as did the perception of thermal comfort during exercise (−0.3 units).

**Conclusion:**

This data evidences the effectiveness of a practical, mixed methods HA strategy, remotely implemented around training and competition, at inducing the heat acclimation phenotype in a high-level soccer match official.

## Background

Heat acclimation (HA) is effective at reducing physiological and thermoregulatory strain before exercise in temperate and hot conditions. The HA phenotype that includes beneficial effects on heat storage, cardiac function and blood distribution has been well documented. Physiologically, noteworthy criteria for successful attainment of HA include decreased resting and exercising, core, skin and whole body temperature, plasma volume expansion and reductions in heart rate at a given exercise intensity, improved sudomotor function, and reduced perception of heat stress. The implementation of an isothermic heat acclimation protocol, whereby the degree of hyperthermia is typically controlled at a rectal temperature ~38.5 °C, has been recommended as a vital component training before competing in the heat (Garrett et al. 2009).

During the 2014 FIFA World Cup, matches scheduled in northern and tropical regions of Brazil posed an increase in the physiological demands due to the hot and humid climate. During an international match, referees cover distances between 9 and 11 km, with high intensity running (>15 km h^−1^) accounting for 5–10 % (0.9–2.39 km). Mean heart rate is between 85 and 90 % of age-predicted maximum heart rate, corresponding to 80 % aerobic capacity (Stølen et al. 2005; Weston et al. 2012). Typically, in northern regions of Brazil, for the month of June, environmental conditions range from 20 °C to 30 °C and 80 % relative humidity (RH). Therefore, based on a body surface area of 2.23 m^2^, mean oxygen uptake of 3 L min^−1^ and RER of 1.00, we estimated mean body heat production to be ≈400 W m^2^ for matches in northern regions of Brazil and predicted a core body temperature ≈38–38.5 °C that would induce considerable cardiovascular and metabolic strain with accompanying increases in perceived exertion and thermal stress (Kenny and Jay 2013). Such combinations impair physical and cognitive performance (Nybo et al. 2014; Qian et al. 2014). Evidence highlights marked changes to soccer performance in the region as a consequence of heat stress, with a reduced number of sprints performed by players (−10 %), compared moderate and low heat stress, and a reduction in the distance covered at a high intensity (−2.1 m min^−1^ player) (Nassis et al. 2015). These alterations likely result from anticipatory pacing to mitigate excessive increases in core and muscle temperature which are known to impair intermittent sprint performance under heat stress (Drust et al. 2005).

To prepare for these conditions, a novel programme that combined active and passive heat exposures to induce HA was designed to improve heat dissipation and attenuate potential decrements in performance. Given the nature of the intervention, which incorporated remote, self-monitored passive session, the data presented in this case report will primarily be of interest to scientists and coaches preparing athletes for training and competing in hot and humid environments, particularly when logistical constraints limit heat-chamber based acclimation programmes.

To maximise the adaptive response, HA should be performed on consecutive days, this, however, can be a challenge to the elite athlete whereby daily access to environmental chambers is problematic. Additionally scheduling HA around structured training and competition can be challenging. In respect, the benefits of a mixed methods HA regime, combining isothermic HA sessions, and passive heat stress after training, provides a strategy whereby the logistical demands of the athlete are reduced but potentiating stimuli for adaptation (repeated, daily increases in core temperature) are maintained.

## Case presentation

The athlete (Age = 43 years, body mass = 96.9 kg, stature = 190 cm) was a professional soccer match official with 11 years’ experience at international level (Weston et al. 2011). During the 2013 Confederations Cup tournament in Brazil 2013, the referee experienced symptoms associated with heat illness which he perceived as negative for health and performance. The athlete requested support to minimise this risk during the 2014 World Cup. The athlete was informed of the risks and discomforts of the proposed sessions and provided written informed consent. The study was approved by the local ethics board and data collection was conducted in accordance with the Declaration of Helsinki (2013).

### Experimental design

The athlete visited the laboratory on eight occasions between 13th May 2014 and 30th May 2014. Prior to each training session the athlete was advised to follow his prescribed diet by his professional organisation and asked to consume at least 500 ml of non-caffeinated fluid 2 h before each session to promote euhydration. During each visit, pre-session assessments of body mass (kg), stature (cm), haematocrit (%) (Sodium heparinised MicroHaematocrit tubes, Hawksley, UK) and haemoglobin (g dL) (HemoCue Hb 201, Radiometer Ltd, UK) (fingertip capillary samples) and urine osmolality (mOsmol kgH_2_O) (Osmocheck, Vitech Scientific Ltd, UK) were conducted. Changes in plasma volume were calculated using the method of Dill and Costill (1974). Towel dried nude body mass was recorded pre and post exercise and used in the assessment of whole body sweat rate.

**Fig. 1:**
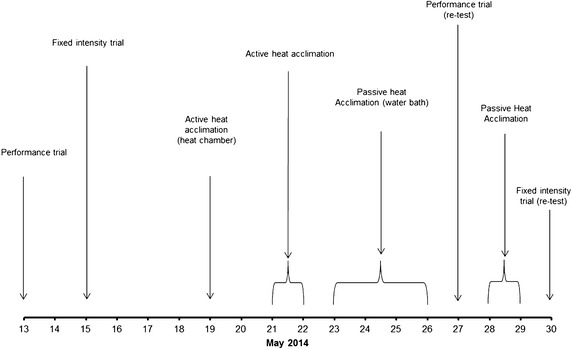
Time-line of scientific support

Figure 1 depicts the time-line of support, to investigate heat acclimation state the athlete performed a heat stress test (HST) and a repeated sprint test, in an environmental chamber set at 30 °C 80 % RH. All tests were performed at the same time of day to minimise the effects of circadian rhythms. To quantify the thermoregulatory and perceptual responses to heat stress, the athlete completed 10 min of exercise at three different intensities (6, 11 and 13.5 km h^−1^) and 5 min at 16 km h^−1^ on a motorised treadmill. Core temperature was assessed at the tympanic (Ty_temp_) membrane (Thermoscan 5, Braun GmbH, Germany) (test re-test typical error = 0.24 °C, coefficient of variation = 0.64 %, assessed in our laboratory). Skin temperature was recorded using U-type thermistors (Grant Instruments, Cambridge, UK), at four sites (chest, bicep, thigh and calf). Expired air was sampled and assessed (Ultima, CardiO_2_, Medgraphics, USA) for 3 min at the end of each stage. Thermal perception was assessed using a 9 point scale (1 very cold–9 very hot) (Nielsen et al. 1989**)**. The repeated high-intensity running test required the athlete to perform as many efforts as possible, up to a maximum of 20 repetitions of 30 s with 30 s passive recovery on a non-motorised treadmill test (Woodway Curve, WI, USA). The aim of the test was to complete all 20 repetitions and cover as much distance as possible. This protocol was implemented due to its similarity to a standardised FIFA refereeing test (Weston et al. 2009).

**Fig. 2:**
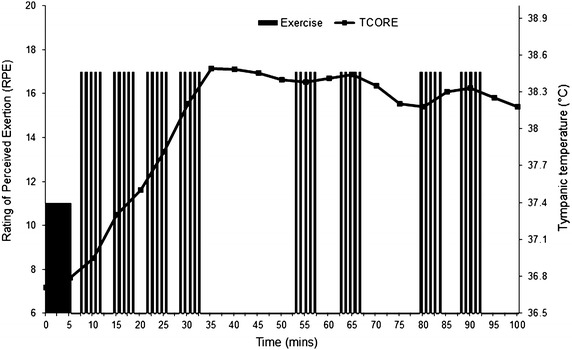
Example of a typical heat acclimation training session involving intermittent sprints. *Solid* column from 0 to 5 min indicates warm up. *Columns* represent sprint efforts

Athlete availability necessitated the use of a novel medium-term HA regime (MTHA 7–10 days) consisting of both isothermic and passive-heat exposures. During the isothermic sessions, tropical environmental conditions (40 °C 80 % RH) were used to limit external heat transfer, increase heat storage and stimulate sudomotor activity. Intermittent high-intensity exercise was performed on a non-motorised treadmill (Woodway Curve, WI, USA) after a 5 min warm-up at a self-selected pace, the athlete performed six, 20-second high-intensity (RPE = 17) efforts followed by 40 s low intensity running (RPE = 11). This protocol was used to simulate refereeing match demands and rapidly increase body temperature providing ecological validity to training. In line with previous work describing the attainment and maintenance of a core temperature ≈38.5 °C, a modified isothermic target core temperature of 38.0 °C measured at the tympanic membrane (Ty_temp_) was maintained, with the 0.5 °C difference reflecting a tendency for tympanic temperature to under-read, relative to rectal (Easton et al. 2007). Upon attaining the target temperature, the number of high-intensity efforts was adjusted to maintain a Ty_temp_ at 38 °C for 60–80 min. A typical session consisted of four to five sets of six, 20 s high-intensity efforts with 40 s passive recovery. The athlete performed 10–15 min of jogging and walking between the sets. When Ty_temp_ exceeded 38 °C the athlete rested (Fig. 2).

To increase physiological strain, we used permissive dehydration and restricted fluid intake to 500 ml per session, to provide a stimulus for plasma volume expansion (Garrett et al. 2014). Passive HA sessions required the athlete to undertake 30 min of intermittent submersion in a hot water bath (≈48 °C) after his typical (temperate condition) high-intensity interval and resistance training sessions. The passive HA sessions involved 30 min whole-body immersion in a hot water bath (≈48 °C) to induce heat strain, similar to the methods of (Scoon et al. 2007; Stanley et al. 2015; Zurawlew et al. 2015). The water bath rapidly increased Ty_temp_ to 38 °C; when Ty_temp_ exceeded 38 °C the athlete exited the bath and rested until Ty_temp_ fell below 38 °C and then re-entered the bath. This intermittent water-bathing protocol lasted 30 min.

### Statistical analysis

Data were assessed for normality and homogeneity of variance. Pre-and post-heat acclimation data were assessed with freely available spreadsheets (http://sportsci.org/resource/stats/xcontrial.xls and http://sportsci.org/resource/stats/xprecisionsubject.xls) using Cohen’s *d* as a measure of effect size and 90 % confidence intervals. In the initial repeated high-intensity running test the athlete reached volitional exhaustion and terminated exercise after 15 efforts, citing a thermal strain of 9 and RPE of 20. Thus, comparison was conducted on the first 15 efforts despite completing 20 sprints post-HA. When the assumption of normality was violated the Wilcoxon Signed Rank test was used. Statistical significance was set at *P* < 0.05.

## Results

**Fig. 3:**
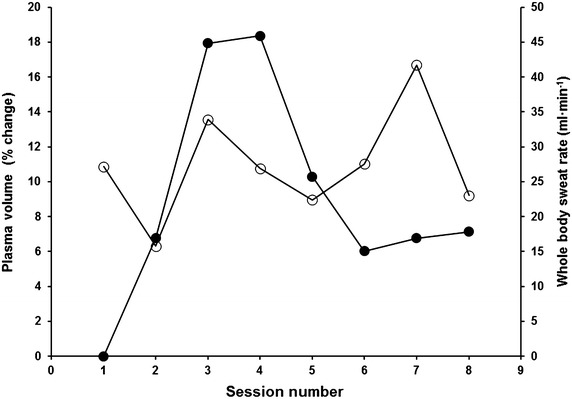
Change in plasma volume (*closed circles*) and sweat rate (*open circles*) across the support period

The mixed methods HA intervention was successful at inducing typical physiological adaptive responses congruous with established methods of preparing individuals for training and competing in the heat (Tables 1 and 2; Fig. 3). The improved physiological and perceptual responses to heat stress contributed to improved high-intensity running performance, a factor known to reduce during soccer performance in conditions of high heat stress.

**Table 1:** Physiological and performance data before and after heat acclimation training

Measure	Pre (±SD)	Post (±SD)	Statistical significance or standardised difference score [*d* (90 % confidence interval)]	Probability (% chance) and qualitative statement
Resting plasma volume (% change)	0	7.1		NA
Sweat rate (L h^−1^)				
Performance trial	1.62	2.52		NA
Fixed intensity trial	0.96	1.38		
Performance trial				
Number of sprints	15	20	NA	NA
Total distance covered (m)	2247	2897	NA	NA
Mean speed (km h^−1^)	18.0 ± 1.0	17.6 ± 0.3	*d* = −0.46 [−1.09 to 0.17]	76 % less
Mean thermal perception	8.5 ± 0.6	8.2 ± 0.4	*P* < 0.05	58 % less
Mean RPE	17 ± 2	17 ± 2	*P* > 0.05	38 % similar
Mean heart rate (beats min^−1^)	175 ± 8	178 ± 7	*d* = 0.31 [−0.32 to 0.95]	66 % greater
Change in Ty_temp_ (°C)	1.8	1.7	NA	50 % less

Data are presented as mean average ± standard deviation (SD) where appropriate

**Table 2:** Physiological and perceptual data during fixed intensity running trial

Measure	Pre (±SD)	Post (±SD)	Statistical significance or standardised difference score [*d* (90 % confidence interval)]	Probability (% chance) that the difference is less, similar to or greater than smallest worthwhile change
*Fixed intensity trial*				
HR mean entire trial (beats min^−1^)	149 ± 36	139 ± 36	*d* = −0.26 [−0.85 to 0.32]	57 % less
HR mean running (beats min^−1^)	169 ± 17	160 ± 17	*d* = −0.54 [−1.23 to 0.15]	79 % less
RPE	13 ± 4	13 ± 3	*d* = −0.07 [−0.69 to 0.54]	41 % similar
$\dot{{V}}$ O2 (L min^−1^)	3.32 ± 0.90	2.88 ± 0.88	*d* = −0.50 [−1.38 to 0.39]	72 % less
RER	1.17 ± 0.09	1.08 ± 0.09	*P* > 0.05	NA
Mean Ty_temp_ (°C)	37.2 ± 0.6	36.6 ± 0.5	*P* < 0.05	87 % less
Change in Ty_temp_ (°C)	1.7	1.9		40 % greater
Resting mean SK_T_ (°C)	34.7 ± 0	33.7 ± 0.1		98 % less
Resting mean core to skin temperature gradient (°C)	1.8	2.2	NA	65 % less
Mean Thermal Perception	7.7 ± 0.7 (“Hot”)	7.4 ± 0.7 (“Warm”)	*P* < 0.05	58 % less

Data are presented as mean average ± standard deviation (SD) where appropriate

### Protocol

High-intensity running, in hot and humid conditions, serves to rapidly increase Ty_temp,_ and might be considered by athletes during tapering or where intermittent sprints are a component of the sport. Ty_temp_ remained above 38 °C for at least 60 min per exercise session and facilitated a mean sweat rate of 2.1 L h^−1^. Our findings of increased sweat rate are supported by research suggesting it takes >12 days to induce adaptations in sudomotor function (Pandolf 1998). During exercise-sessions we employed isothermal strain and permissive dehydration to restrict fluid intake to 500 ml per session. This resulted in a mean body mass loss of 2 % similar to previous research (Garrett et al. 2014) and is a potential explanation for plasma volume expansion.

Unfortunately, we were unable to report the training load during the intervention but the athlete reported that he maintained typical training and refereeing requirements. The intervention was associated with several risks resulting from high body temperature and permissive dehydration which were increased during the self-managed water baths. However, these were controlled using Ty_temp_ and the athlete reported no adverse effects. Furthermore, by using tympanic temperature and thermal perception we were able to control body in a practical manner to induce heat acclimation. Therefore, as well as future research investigating the combination of active and passive heat exposure, the control of body temperature to induce acclimation using the practical method of Ty_temp_ versus rectal or oesophageal temperature might be of interest to applied scientists.

### Physiological responses

We observed physiological responses typical of heat acclimation (Sawka et al. 2011). Heart rate was less during submaximal exercise, likely modulated by the observed plasma volume expansion. The passive heat stress element of the mixed methods HA protocol used in this study is comparable to previous research (Stanley et al. 2015; Zurawlew et al. 2015), that used a sauna (87 ± 13.7 °C) and water bathing (40 °C) to impose heat stress for 30 min a day for 10 and 6 days, respectively. Similar to our data, plasma volume increased by 7 % after 8 days of heat exposure in Stanley et al. (2015) and 3 ± 5 % after 6 days in Zurawlew et al. (2015). However, only trivial changes in HR during exercise were observed in Stanley et al. (2015) whereas we noted a 10 beats min^−1^ decrease in mean HR, similar to the 6 (90 % confidence interval 2–10) beats min^−1^ reported by Zurawlew et al. (2015). Ty_temp_ was less at rest and during exercise, accompanied by a lower mean skin temperature at rest similar to the changes observed by Zurawlew et al. (2015), but skin temperature was not different during exercising. However, this might be due to a reduced core temperature threshold for cutaneous vasodilation, evidenced by a decrease in core-to-skin temperature gradient, an index of skin blood flow (Kenefick et al. 2007). Sweat rate increased in-line with that reported by Zurawlew et al. (2015), and was likely accompanied by an earlier onset of sweating and decreases in sweat sodium concentration (Chinevere et al. 2008). Oxygen uptake and respiratory exchange ratio, indicative of substrate metabolism, reduced during exercise, potentially attenuating metabolic heat production and preserving muscle glycogen. Perception of thermal comfort improved, but RPE was similar. These beneficial adaptations likely underpin the improved running performance.

### Performance

The athlete increased distance covered in the repeated high-intensity running test by 29 %, which is similar relative improvement to a 32 % decrease in 5 km run time observed after 3 weeks of post-training 30 min saunas (Scoon et al. 2007) and greater than the 4.9 % improvement reported by Zurawlew et al. (2015). We acknowledge the possibility of a learning effect occurring in both the repeated high-intensity running and fixed intensity tests after the intervention. However, the likely beneficial changes to a number of physiological variables infer that this was unlikely. Indeed, despite the low internal-validity of this study, the athlete acquired phenotypical responses associated with heat acclimation despite possible interactions with other training and circadian variables.

### Practical applications

These results are of interest to practitioners who have limited access to hot environments or climatic chambers in which to prepare their athletes. We have demonstrated a novel approach whereby the athlete completes high intensity, sport-specific training in an environmental chamber, before safely and conveniently completing passive heat-stress sessions remotely. In this manner, the use of tympanic temperature monitoring offers a cost effective, practical and simple method for athlete’s to safely self-monitor remote passive sessions. Such an approach may be of particular interest when a large group of athletes require preparation, however practitioners should be aware that at the start of heat acclimation training there might be a transient decrease in physical capacity, which might influence training periodisation. We do however recommend that athletes are suitably familiarised with procedures in order to accurately self-monitor tympanic temperature.

## Conclusion

This is the first evidence that a novel mixed methods HA regime that combined active and passive heat exposures over a period of 18 days. The protocol was sufficient to induce physiological and thermoregulatory adaptations that contributed to improved high-intensity running performance in the heat. High intensity sprinting is an ecologically valid and effective method to increase core temperature in accordance with isothermic heat acclimation strategies. In addition, when financial or logistical constraints limit heat-chamber induced acclimation the inclusion of short-periods of hot-water immersion might be a useful complimentary heat-stress stimulus.

## Availability of data and materials

Data sets are available from the corresponding author on request.
